# (Eco)Toxicology of Cyanobacteria and Cyanotoxins: From Environmental Dynamics to Adverse Effects

**DOI:** 10.3390/toxics10110648

**Published:** 2022-10-28

**Authors:** Mauro Vilar, Aloysio Ferrão-Filho

**Affiliations:** 1 Laboratory of Ecophysiology and Toxicology of Cyanobacteria, Carlos Chagas Filho Institute of Biophysics, Federal University of Rio de Janeiro, Carlos Chagas Filho Avenue, Rio de Janeiro 21949-902, RJ, Brazil; 2 Laboratory of Evaluation and Promotion of Environmental Health, Instituto Oswaldo Cruz, FIOCRUZ, Brasil Avenue, 4365, Manguinhos, Rio de Janeiro 21045-900, RJ, Brazil

The problem of artificial eutrophication, together with the effects of climate changes has led to an increase in the frequency of the occurrence of cyanobacterial blooms. In fact, these factors have been pointed out as intensifying the proliferation of these photosynthetic microorganisms in several water bodies worldwide [1]. In addition, cyanobacteria pose considerable public and environmental health risks, as some species are potential producers of bioactive secondary metabolites called cyanotoxins. These secondary metabolites are biochemically classified as cyclic peptides (hepatotoxins), organophosphates (neurotoxin), and alkaloids (neuro- and cytotoxins) [2,3].

Among bioactive peptides are nodularin (NOD) and microcystins (MCs) as the most studied cyanotoxins, comprising cyclic peptides referred to as hepatotoxins due to their damage to hepatocytes in mammals. These peptides are potent serine–threonine phosphatase enzyme inhibitors which comprise more than 300 variants [4] according to structural variations resulting from substitutions and demethylations in some of the amino acids in positions 2 and 4 and 3 and 7, respectively [5]. MCs and NOD can accumulate in various aquatic organisms from invertebrates such as zooplankton and aquatic insects to vertebrates such as fish, reptiles, birds, and mammals [6,7,8]. In general, MCs and NOD can reach and accumulate in the tissues via the ingestion of cyanobacterial cells [7,8,9], contaminated food [10], or directly via the absorption of dissolved toxins from the water [11,12,13]. The most important incorporation route is, however, through transference along the foodweb [8,9,14,15]. In spite of its transference potential, MCs tend not to biomagnify but rather biodilute in the foodweb [6,8,16]. Both cyclic peptides are relatively stable in a wide range of temperatures and pH levels and are not efficiently removed by conventional water treatments [17,18,19]. In addition, MCs can be adsorbed to natural organic matter and lake sediments and are naturally degraded in water through photolysis and biodegradation by indigenous microbial populations [19,20].

Furthermore, bioactive alkaloid compounds are among the most potent natural toxins, with saxitoxins and cylindrospermopsins standing out. Saxitoxins (STXs) constitute about 56 variants of neurotoxic guanidine alkaloids that act to block ion channels (Na^+^ and Ca^2+^) and modulate the blocking pattern of potassium (K^+^) channels in cells [21]. On the other hand, cylindrospermopsin (CYN) and its analogues are tricyclic guanidine alkaloids with cytotoxic potential, which inhibit protein synthesis and are considered genotoxic (covalent binding to nucleic acids) and may also cause oxidative damage [22]. STXs and CYNs are produced by several species of bloom-forming cyanobacteria, among which *Raphidiopsis raciborski* stands out due to its wide distribution (and invasive potential), adaptive plasticity, predominance, and therefore, contribution to the occurrence of these toxic alkaloids in different water bodies [23], with South American strains being reported as saxitoxin producers only [24,25]. Due to the high stability of these cyanotoxins, even after the decay of blooms, these metabolites can persist in the environment. Although the stability of saxitoxin and its analogues is pH- and temperature-dependent [26], these toxins can exhibit a half-life of about 9 to 28 days at neutral pH and room temperature (~25 °C) in natural waters [27]. CYNs are more resistant, showing stability under varying pH and temperatures and have been reported to persist in water for over a month [28]. CYNs are predominantly extracellular, occurring in high concentrations in the dissolved fraction and accounting for 20–99% of the total CYN content of the bloom [29,30]. Therefore, the prevalence of these toxins and their high solubility in water (high polarity) represent a considerable health risk, especially when these water sources are used for public supply, recreational activities, and animal drinking. 

Anatoxins (ATXs) are also among the neurotoxic alkaloids produced by cyanobacteria, together with the organophosphate guanitoxin (GNT) (formerly anatoxin-a(S); [31]). Although they can occur around the world, ATXs and GNT have been detected less frequently than MCs, STXs, and CYNs [18]. ATX-a is highly soluble in water and stable under acidic conditions but is unstable in an alkaline medium and degrades rapidly in sunlight and temperature above 20 °C [31]. Its biodegradation has been reported as performed by bacteria and protozoa from sediments [32]. Little is known about the persistence and degradation of GNT in the environment, probably because it is an unstable compound [4,32]. While ATXs’ mechanism of action is through binding to nicotinic acetylcholine receptors of nerve cells, GNT binds irreversibly to acetylcholinesterase in the neuro-muscular junctions, blocking the degradation of acetylcholine. Both toxins lead to nerve hyperexcitability, causing overstimulation of muscles, respiratory distress (dyspnoea) and convulsions preceding death, which occurs due to respiratory arrest [18]. Neurotoxic effects in dogs and livestock, resulting occasionally in death, has been reported after ingestion of water containing high densities of ATX-producing cyanobacteria [18].

Nevertheless, cyanobacteria have displayed a range of metabolites whose biological activity is still incipient. More recently, Jones et al. [4] presented the CyanoMetDB: a database of cyanometabolites encompassing more than 2000 molecules. The authors also have included more than 300 microcystin variants, which highlights the need for investigating the effects of these frequently reported bioactive peptides. In addition, another neurotoxic cyanometabolite named aetokthonotoxin (AETX) and known as “eagle killer” has been recently elucidated and reported as the cause of vacuolar myelinopathy in North American bald eagles [33]. The study also identified a novel cyanobacterial species (*Aetokthonos hydrillicola*) as the AETX producer, which curiously depends on the availability of bromide for the toxin’s biosynthesis, occurring as a periphytic cyanobacteria in submerged macrophytes such as those from the genus *Hydrilla*.

Although cyanobacterial metabolites pose serious hazard to animals and humans by themselves, the co-occurrence of cyanobacteria and cyanotoxins in combinations with other stressors, including algal toxins, microbial pathogens, metals, pesticides, nanoparticles, pharmaceuticals and microplastics, pose additional threats in the environment [34]. Nanoparticles and microplastics, for example, can adsorb cyanotoxins, serving as a vector of these toxins to organisms that ingest these particles [35,36,37]. Other stressors can interact with cyanotoxins, causing additive, synergistic, or even antagonistic effects to the biota [34,38].

In the last four decades of research on cyanobacteria and their metabolites, huge advancements have been accomplished, and several questions have been answered regarding their physiology, toxicology, and dynamics in the environment. Nevertheless, new challenges have emerged, especially due to global climate changes, with the need to predict the fate and consequences of anthropogenic disturbance in aquatic ecosystems under a warmer and more polluted scenario. For this reason, this Special Issue aims to present research and review papers that address the dynamics and effects of harmful cyanobacteria and their bioactive metabolites from the individual to ecosystem level, encompassing the study of well-known cyanotoxins and less-investigated or novel cyanometabolites. As guest editors, we especially encourage advances and novelties regarding cyanotoxins analysis and monitoring, mainly on their fate in freshwater and coastal environments (e.g., bioaccumulation, biodegradation); toxicological and ecotoxicological assessments of single toxins and their mixture with other hazardous substances; and the chemical, eco-physiological, and molecular characterization of isolated strains or natural populations of harmful cyanobacteria.
